# Low NLRP3 expression predicts a better prognosis of colorectal cancer

**DOI:** 10.1042/BSR20210280

**Published:** 2021-04-16

**Authors:** Feng Shi, Bo Wei, Tian Lan, Yang Xiao, Xin Quan, Jun Chen, Chong Zhao, Jinhang Gao

**Affiliations:** 1 Lab of Gastroenterology and Hepatology, West China Hospital, Sichuan University, Chengdu, China; 2 Key Laboratory of Brain Science Research of Hainan Province, Hainan Medical University, Haikou, China; 3 Department of Gastroenterology, West China Hospital, Sichuan University, Chengdu, China; 4 Department of Human Anatomy, West China School of Basic Medical Sciences and Forensic Medicine, Sichuan University, Chengdu, China

**Keywords:** colon adenocarcinoma, inflammasome, interleukin 1β, interleukin 18, Survival analysis

## Abstract

**Background:** NOD-like receptor pyrin domain-3 (NLRP3) inflammasome activation is a double-edged sword in tumorigenesis. Whether NLRP3 is involved in the progression and prognosis of colorectal cancer (CRC) remains elucidated and is the focus of the present study.

**Methods:** Immunohistochemistry (IHC) was applied on tissue microarray (TMA) to determine the expression of NLRP3 in CRC patients. All 100 patients were divided into the low NLRP3 group and the high NLRP3 group according to their NLRP3 IHC scoring. Additionally, CRC xenografts were established by injecting HCT116 or RKO cells subcutaneously into nude mice. Cell proliferation and apoptosis were determined in HCT116 cells after treatment with NLRP3 inhibitor MCC950.

**Results:** NLRP3 expression was up-regulated in colon adenocarcinoma tissues compared with that in paracancerous tissues in CRC patients, HCT116 xenograft, and RKO xenograft. High NLRP3 level correlated with the advanced TNM classification of malignant tumors, the occurrence of distant metastasis, vascular invasion, and positive lymph nodes. Furthermore, Kaplan–Meier survival analysis revealed that a high NLRP3 level was associated with a low 5-year survival rate and even a low 10-year survival rate. Moreover, the multivariable Cox proportional hazards regression model implied that NLRP3 expression level was an independent risk factor for CRC prognosis. Inhibition of NLRP3 by MCC950 suppressed cell proliferation, induced cell apoptosis, and decreased mRNA levels of interleukin 1β (IL1β) and interleukin 18 (IL18) in HCT116 cells.

**Conclusions:** High level of NLRP3 predicts poor survival in CRC patients. NLRP3 is a putative prognostic biomarker and a potential therapeutic target in CRC treatments.

## Introduction

Colorectal cancer (CRC) is the third most common cancer in China and accounting for nearly 200000 deaths per year in China [1]. The stage at diagnosis is the most important prognostic factor. The 5-year survival rate is 90% when CRC is detected at an early stage, while the average survival rate is 65% for CRC patients [1–3]. From 2000 to 2017, death rates declined annually, and the death rate of CRC has decreased by 34% among individuals older than 50 years [2,4]. However, the clinical outcome is still unsatisfactory due to the poor implementation of CRC screen tests and lack of a single risk factor for CRC progression and prognosis [5,6]. Consequently, it is of great importance to discover practical risk factors for CRC patients.

Inflammasomes are a multiprotein complex that can trigger the innate immune reaction, inflammation, and pyroptotic cell death in response to damage- and pathogen-associated molecular patterns (DAMPs and PAMPs) [7]. According to their major component, inflammasomes can be classified as AIM2, NLRC4, NLRP1, and the best-characterized NOD-like receptor pyrin domain-3 (NLRP3) inflammasome [8,9]. NLRP3 inflammasome contains the NLRP3, the adaptor protein apoptosis-associated speck-like protein containing a C-terminal caspase recruitment domain (ASC) and the effecter protein pro-caspase-1. NLRP3 recruits ASC to cleave and activating pro-caspase-1 and then provokes and spreads robust inflammation. The primary function of the NLRP3 inflammasome is to inducing cell pyroptosis via cleaving Gasdermin D (GSDMD) and to enhancing inflammation by releasing interleukin 1β (IL1β) and interleukin 18 (IL18) [7,10]. It has been demonstrated in the previous studies that the NLRP3 inflammasome and its downstream products are involved in the carcinogenesis of many cancers, including colitis-associated cancer, lung cancer, breast cancer, and melanoma [7,11,12]. In terms of CRC, it has been reported that increased susceptibility of dextran sulfate sodium (DSS)-induced colitis and colitis-associated tumorigenesis in NLRP3^−/−^ and IL18^−/−^ mice, respectively [13,14]. Consistently, NLRP3 activation can reduce the initiation of colitis-associated cancer [15]. However, a portion of studies indicates NLRP3 inflammasome activation as a double-edged sword in tumorigenesis [7,16]. Except for the debut in the exact effect of NLRP3 in tumorigenesis, the correlation between NLRP3 and the prognosis of CRC remains unclear.

The present study aims to evaluate the correlations between NLRP3 expression and the prognosis of CRC. We found that high expression of NLRP3 is associated with poor survival and prognosis in colon adenocarcinoma. Inhibition of NLRP3 by MCC950 may suppress cell proliferation and induces cell apoptosis via inhibiting IL1β and IL18.

## Materials and methods

### Tissue microarray

Human CRC tissue microarray (TMA) was obtained from National Engineering Center for Biochip at Shanghai (Shanghai, China). All 100 patients had undergone complete surgical resection of the colon adenocarcinoma between July 2005 and December 2010. Of which, 60 corresponding paracancerous tissues were also collected. Additionally, colon cancer and corresponding paracancerous frozen tissues were also collected from eight patients diagnosed with colon cancer in West China Hospital. Written informed consent forms were received from all patients involved in the present study. The present study was approved by the Ethical Committee of Biobank Center Related Hospitals. The clinicopathological information of all patients was listed in Table 1. Follow-up for all patients was completed from the date of surgery until September 2015.

**Table 1:** Clinicopathological characteristics of 100 colon adenocarcinoma cases

Characteristics	Number of cases	Percentage (%)
Total	100	100
Average years	62.4 ± 11.6	
<65	54	54.0
≥65	46	46.0
Gender		
Male	58	58.0
Female	42	42.0
Histologic grade		
I–II	78	78.0
II–III	22	22.0
TNM stage		
I	3	3.0
II	58	58.0
III	38	38.0
IV	1	1.0
Location (cm)		
Left colon	42	42.0
Right colon	58	58.0
Infiltration degree		
Adventitia	70	70.0
Serosa/muscular/mucosa	30	30.0
Pathological morphology		
Infiltrate/ulcer type	75	75.0
Protrude/basin type	25	25.0
Distant metastasis and vascular invasion	18	18.0
Positive lymph nodes	38	38.0

### Cell culture and treatments

Human colon adenocarcinoma cell HCT116 and RKO was obtained from the Type Culture Collection of the Chinese Academy of Sciences (Shanghai, China). HCT116 and RKO cells were cultured in RPMI-1640 (HyClone, Logan, UT, U.S.A.) and DMEM (HyClone), respectively. The medium was supplemented with 10% fetal bovine serum (Gibco, Logan, UT, U.S.A.), 100 U/ml penicillin, and streptomycin (HyClone). Cells were cultured in the incubator at 37°C with 5% CO_2_. HCT116 and RKO cells were trypsinized and replated when cell density reached 80–90%.

### Cell counting kit-8 (CCK8) assay for cell viability

HCT116 cells were seeded at a density of 10^4^ cells/well in 96-well plates for 24 h. Then the cells were treated with DMSO or MCC950 (2 μM) for 24 h. Ten microliters of CCK8 solution (Dojindo, Kumamoto, Japan) was then added and incubated at 37°C for 1 h in the dark. The optical density was measured at 450 nm using the Thermo microplate reader (Thermo Fisher Scientific, Waltham, MA, U.S.A.).

### Flow cytometric analysis for cell cycle and apoptosis

HCT116 were seeded in six-well plates and treated with DMSO or MCC950 (2 μM) for 24 h. For cell cycle detection, the cells were collected and incubated with RNase A at 37°C for 30 min and stained with Propidium Iodide (PI, Keygen Biotech, Nanjing, China) at 4°C for 30 min in the dark. The cell cycle data were collected by flow cytometry (CytoFLEX, Beckman Coulter, Indianapolis, IN, U.S.A.) and analyzed using ModFitLT5.0 software (Verity SoftwareHouse, Topsham, ME, U.S.A.).

Cell apoptosis of HCT116 was examined by using an AnnexinV-FITC/PI dual staining kit (BD Biosciences, San Jose, CA, U.S.A.) according to the manufacturer’s instructions [17]. Briefly, the cells were collected and stained with Annexin V-FITC and PI in 400 μl of binding buffer in the dark at room temperature for 15 min and quantified by flow cytometry (CytoFLEX).

### Human CRC xenografts

Twelve nude mice, weighing 18–22 g, were obtained from the Experimental Animal Center of Sichuan University (Chengdu, China). All mice were kept under 12 h light–dark cycles at a constant temperature and humidity with free access to chow and water. Mice were randomly separated into two groups, with six mice in each group according to the body weight. One group received a subcutaneous injection of 1 × 10^7^ HCT116 cells, while another group received a subcutaneous injection of 1 × 10^7^ RKO cells. Xenograft mice were killed under anesthesia by overdose sodium pentobarbital via intraperitoneal after 4 weeks. Subcutaneous colon adenocarcinoma tissues and normal colons were collected and fixed in 4% neutral buffered paraformaldehyde for histopathologic and immunohistochemical staining. The animal procedures were approved by the Animal Use and Care Committee of Sichuan University and were conducted according to regulations set by Sichuan University.

### Hematoxylin–Eosin (H&E) staining

Tissues fixed in 4% paraformaldehyde were embedded in paraffin, sectioned (thickness of 6 μm), and then stained with H&E. The morphological changes were examined under a microscope (CX41, Olympus, Tokyo, Japan) equipped with a digital camera (DP72, Olympus).

### Immunohistochemistry (IHC)

IHC was applied to determine the expression of NLRP3 in tissue samples gained from CRC patients and xenograft mouse models. Briefly, the TMA section was deparaffinized in xylene and rehydrated with graded ethanol dilutions. Antigen retrieval was performed by heating the sections in 10 mM sodium citrate buffer (pH = 6.0). Then the sections were blocked by 3% hydrogen peroxide, followed by incubation at 4°C overnight with rabbit anti-NLRP3 (Proteintech, Wuhan, China). After incubation with horseradish peroxidase (HRP)-conjugated secondary antibody kit (ZSGB Bio, Beijing, China) at room temperature, sections were stained by 3,3′-diaminobenzidine tetrahydrochloride and counterstained with Hematoxylin.

### IHC scoring

Human TMA was assessed by two pathologists blind to the clinicopathological information of the patients. For human TMA, a specific semi-quantitatively score system based on positive percentage and intensity of stained has been described previously [18–20]. The positive percentage of stained tumor cells was defined as: 0, no staining; 1, <20%; 2, 20–75%; 3, >75%. The intensity of stained tumor cells was scored on the following scale: 0, negative; 1, weak; 2, moderate; 3, strong staining. The staining index = positive percentage × intensity score. Based on the staining index, a final total score of 0–4 was considered a low expression of NLRP3, whereas a total score of 5–9 was defined as a high expression of NLRP3. Additionally, the tissue samples of the mouse model were scored by the integral optical density (IOD) scoring system according to the staining intensity of NLRP3 by Image-Pro Plus 4.0 software (Media Cybernetics, Silver Spring, MD, U.S.A.).

### Western blot analysis for NLRP3 protein expression

Protein extraction was performed for cultured cells, frozen human, and mouse tissues with a protein extraction kit (Nanjing Kaiji, Nanjing, China). The same amount of protein (50 μg) from each sample was analyzed by gel electrophoresis and transferred respectively to PVDF membrane (Millipore, Billerica, MA, U.S.A.). PVDF membranes were treated with 5% non-fat dry milk and then incubated with primary antibodies directed against glyceraldehyde 3-phosphate dehydrogenase (GAPDH, 1:5000, Abcam, Cambridge, U.K.), NLRP3 (1:2000, Proteintech) at 4°C overnight. After washing, membranes were incubated with appropriate horseradish-peroxidase-conjugated secondary antibodies (1:10000, Santa Cruz) for 2 h at 37°C. The bands were then visualized with an ECL detection kit (Engreen, Beijing, China), determined by Quantity One software 4.5.0 (Bio-Rad, Hercules, CA, U.S.A.), and normalized to GAPDH.

### Quantitative real-time PCR

Total RNA from HCT116 cells was extracted by TRIzol reagent (Invitrogen, Carlsbad, CA). cDNA was synthesized by using SuperScript IV Reverse Transcriptase (Thermo Fisher, U.S.A.). Quantitative real-time PCR (qRT-PCR) analysis of IL1β, IL18, and internal control β-Actin was performed using SYBR® Premix ExTaq™ II (Takara, Dalian, China). The primers included: human IL1β forward: 5′-GCACCTTCTTTCCCTTCAT-3′, reverse: 5′-ACACCACTTGTTGCTCCATA-3′; human IL18 forward: 5′-GATAGCCAGCCTAGAGGTATGG-3′, reverse: 5′-CCTTGATGTTATCAGGAGGATTCA-3′; human β-Actin forward: 5′-GAAGAGCTACGAGCTGCCTGA-3, reverse: 5′-CAGACAGCACTGTGTTGGCG-3′. The cycle program was as follows: 95°C for 5 min followed by 40 cycles of 95°C for 30 s and 60°C for 30 s on a CFX connect PCR Thermocycler Instrument (Bio-Rad, Hercules, CA, U.S.A.).

### Statistical analysis

All data were expressed as mean ± standard deviation and analyzed using SPSS 19.0 software (SPSS, Chicago, IL, U.S.A.). Student’s *t* test was applied for two groups comparing quantitative data. Survival curves were evaluated by using the Kaplan–Meier method and compared by the log-rank test. The χ^2^ test was applied to analyze the difference of clinicopathological parameters between the low and the high NLRP3 groups. Multivariable Cox proportional hazards regression model was applied to determine hazard ratios of overall survival. A *P*-value of <0.05 was considered statistically significant.

## Results

### Patients’ characteristics

The clinicopathological characteristics of 100 colon adenocarcinoma cases is summarized in Table 1. Among 100 cases, the average age were 62.4 ± 11.6, with 54 cases younger than 65 years and 46 cases older than 65 years. Fifty-eight of them were male, and 42 were female. In histological grade, 78 of 100 cases were stage I–II, while 22 of 100 cases were stage II–III. Moreover, the number of cases identified as TNM classification of malignant tumors (TNM) stage I, II, III, IV were 3, 58, 38, 1, respectively. With respect to location, 42 cases were located in the left colon, and the other 58 cases were located in the right colon. Regarding infiltration degree, 70 cases had been infiltrated to the adventitia, and 30 cases had been infiltrated to serosa, muscular, or mucosa. For pathological morphology, 75 cases were infiltrated and/or ulcer type, and 25 cases were protruding or basin type. Among 100 cases, 18 of which had occurred distant metastasis and vascular invasion, and 38 cases were observed with positive lymph nodes.

### NLRP3 up-regulated in colon adenocarcinoma

**Figure 1:**
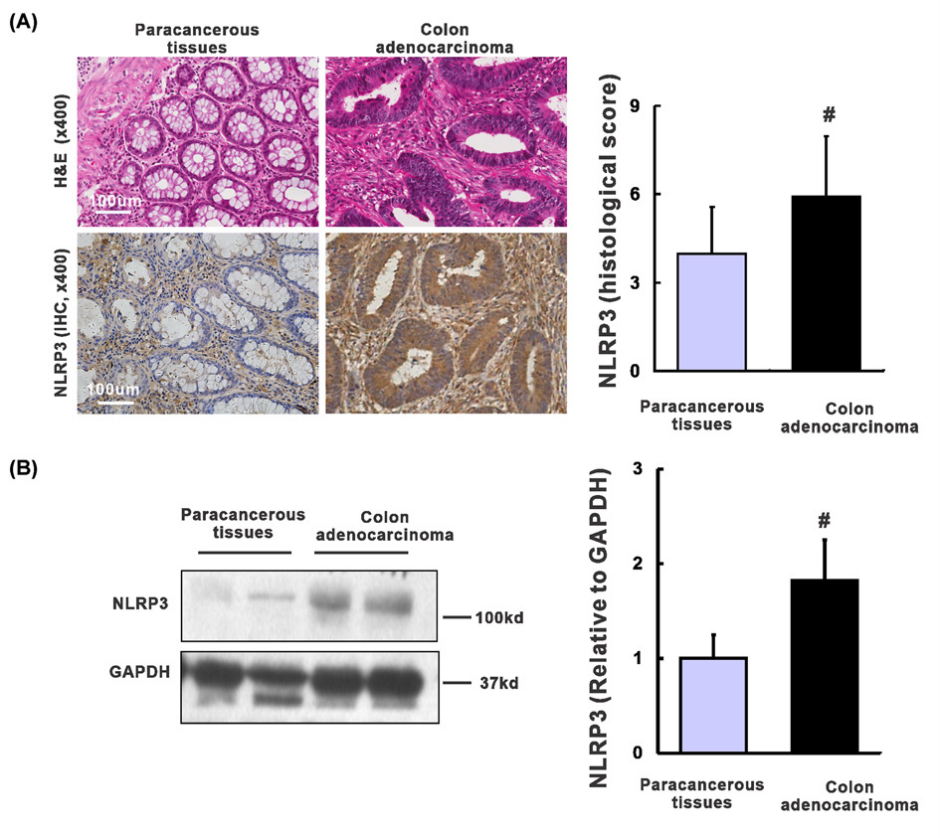
Up-regulation of NLRP3 in human colon adenocarcinoma tissues (**A**) The H&E staining and IHC staining of NLRP3 expression in human colon adenocarcinoma tissues and paracancerous tissues, and quantification of IHC based on the histological score, *n*=60. (**B**) Western blot and quantification of NLRP3 in human colon adenocarcinoma tissues and paracancerous tissues, *n*=8/group. ^#^*P*<0.05 *vs*. paracancerous tissues.

Sixty pairs of human TMA cases were analyzed to assess the expression level of NLRP3 in human colon adenocarcinoma tissues and paracancerous tissues. Representative H&E staining shows irregular tubular structures formed by CRC cells in colon adenocarcinoma tissues compared with typical colon structures in corresponding paracancerous tissues (Figure 1A). A high positive rate of NLRP3 staining in immunocytes and little positive staining of colon epithelial cells was observed in paracancerous tissue (Figure 1A). However, in colon adenocarcinoma tissues, intense positive staining of NLRP3 was found in the cytoplasm of CRC cells (Figure 1A). Compared with that in corresponding paracancerous tissues, the expression of NLRP3 in colon adenocarcinoma tissues was higher in 49 cases and lower in 11 cases. Importantly, based on the staining index, a significant up-regulation of NLRP3 expression was found in colon adenocarcinoma tissues compared with corresponding paracancerous tissues (Figure 1A, 5.6 ± 2.7 *vs*. 3.7 ± 1.8, *P*<0.01). Similarly, the protein level of NLRP3 quantified by Western blot was remarkably increased in colon adenocarcinoma tissues compared with corresponding paracancerous tissues (Figure 1B, 1.82 ± 0.34 *vs.* 1.00 ± 0.24, *P*=0.023).

**Figure 2:**
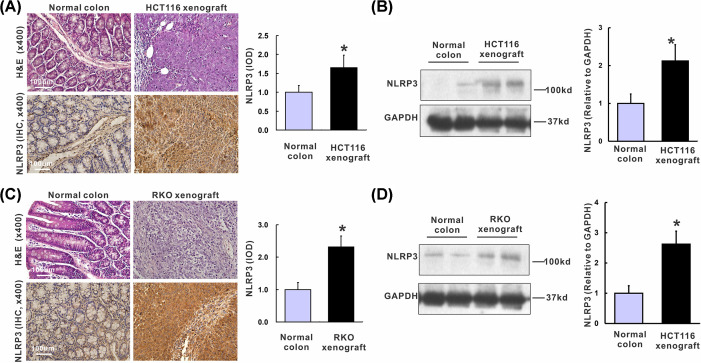
Up-regulation of NLRP3 in human colon adenocarcinoma xenografts (**A**) H&E staining, IHC staining of NLRP3 in HCT116 xenografts and normal colons in nude mice, and quantification based on IOD. (**B**) Western blot and quantification of NLRP3 in HCT116 xenograft tissues and normal colons in nude mice. (**C**) H&E staining, IHC staining of NLRP3 in RKO xenografts and normal colons in nude mice, and quantification based on IOD. (**D**) Western blot and quantification of NLRP3 in RKO xenografts and normal colons in nude mice. *n*=6/group, *P<0.05 *vs*. normal colons.

Next, we evaluated the expression of NLRP3 in HCT116 and RKO xenografts nude mice. Representative H&E staining showed normal morphology and structure in the normal colon, whereas HCT116 and RKO xenografts displayed a poorly differentiated adenocarcinoma histological change (Figure 2A,C). Consistent with the human TMA results, positive staining of NLRP3 was mainly observed in immunocytes in the normal colon compared with that mainly located in the cytoplasm of CRC cells in HCT116 and RKO xenografts (Figure 2A,C). IOD results further confirmed that NLRP3 was significantly higher in colon adenocarcinoma tissues compared to the normal colons in both HCT116 xenografts (Figure 2A, 1.95 ± 0.43 *vs.* 1.00 ± 0.28, *P*=0.035) and RKO xenografts (Figure 2C, 2.52 ± 0.43 *vs.* 1.00 ± 0.25, *P*=0.003). Consistently, the protein level detected by Western blot revealed a similar pattern that NLRP3 was substantially increased in colon adenocarcinoma tissues compared with the normal colon in both HCT116 xenografts (Figure 2B, 2.12 ± 0.35 *vs.* 1.00 ± 0.14, *P*=0.018) and RKO xenografts (Figure 2D, 2.62 ± 0.43 *vs.* 1.00 ± 0.17, *P*=0.011). In summary, NLRP3 is up-regulated in colon adenocarcinoma.

### NLRP3 expression correlated with progression of human colon adenocarcinoma

To explore the correlation between NLRP3 expression and clinicopathological characteristics, we separated the aforementioned 100 patients into two groups according to their NLRP3 histological score. Thirty-three patients were included in the low NLRP3 group and 67 patients in the high NLRP3 group. Then statistical analysis was applied to reveal whether NLRP3 expression level correlated with clinical factors. As presented in Table 2, TNM stage (*P*=0.004), the occurrence of distant metastasis and vascular invasion (*P*=0.030), and positive lymph nodes (*P=*0.005) were found to correlate with NLRP3 expression. Patients in the high NLRP3 group had a lower rate of early TNM stage (I or II) than that in the low NLRP3 group (50.7% *vs*. 81.8%). On the other hand, the rate of advanced TNM stage (III or IV) in the high NLRP3 group was much higher than that in the low NLRP3 group (49.3 *vs*. 18.2%,). It indicates that high NLRP3 expression has a strong positive correlation with the advanced TNM stage. Similarly, high NLRP3 expression also indicated more frequency in the occurrence of distant metastasis and vascular invasion (23.9 *vs*. 6.1%) and positive lymph nodes (47.8 *vs*. 18.2%) compared with that in the low NLRP3 group. However, other clinicopathological features, such as age, gender, histological grade, location, infiltration degree, and pathological morphology, did not present a significant correlation with NLRP3 expression (*P*>0.05). In summary, high expression of NLRP3 predicts advanced TNM stage, the occurrence of distant metastasis, and vascular invasion, and positive lymph nodes in human colon adenocarcinoma.

**Table 2:** Relationships between NLRP3 expression and clinicopathological characteristics in 100 colon adenocarcinoma cases

Characteristics	NLRP3 expression (%)		*P*-value
	Low	High	
Total	33	67	
Average years	61.9 ± 9.9	62.7 ± 12.5	
<65	22 (66.7)	32 (47.8)	0.090
≥65	11 (33.3)	35 (52.2)	
Gender			
Male	20 (60.6)	38 (56.7)	0.830
Female	13 (39.4)	29 (43.3)	
Histologic grade			
I–II	26 (78.8)	52 (77.6)	1.000
II–II	7 (21.2)	15 (22.4)	
TNM stage			
I–II	27 (81.8)	34 (50.7)	0.004
III–IV	6 (18.2)	33(49.3)	
Location (cm)			
Left colon	14 (42.4)	28 (41.8)	1.000
Right colon	19 (57.6)	39 (58.2)	
Infiltration degree			
Adventitia	21(63.6)	49 (73.1)	0.360
Serosa/muscular/mucosa	12 (36.4)	18 (26.9)	
Pathological morphology			
Infiltrate/ulcer type	24 (72.7)	51 (76.1)	0.807
Protrude/basin type	9 (27.3)	16 (23.9)	
Distant metastasis and vascular invasion	2 (6.1)	16 (23.9)	0.030
Positive lymph nodes	6(18.2)	32 (47.8)	0.005

### High expression of NLRP3 associated with poor survival in colon adenocarcinoma

**Figure 3:**
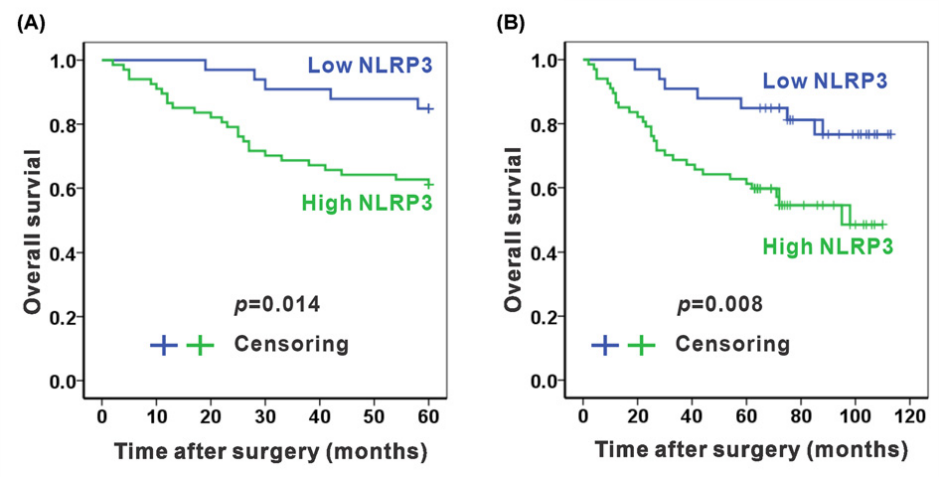
High NLRP3 expression predicted poor survival in human colon adenocarcinoma (**A**) High NLRP3 expression is associated with poor 5-year overall survival. (**B**) High NLRP3 expression is also associated with poor 10-year overall survival. *n*=33 and 67 in the low NLRP3 group and the high NLRP3 group, respectively.

Kaplan–Meier survival analysis revealed that 5-year overall survival after surgery in the high NLRP3 group was significantly poor than that in the low NLRP3 group (Figure 3A, *P*=0.014). The average survival time was 45.5 months for the high NLRP3 group and 56.3 months for the low NLRP3 group. Additionally, the 5-year survival rates were 61.2 and 84.8% in patients with high NLRP3 group and low NLRP3 group, respectively. Consistently, 10-year overall survival was also unfavorable in the high NLRP3 group than that in the low NLRP3 group (Figure 3B, *P*=0.008). The average 10-year survivals were 72.6 and 98.6 for the high NLRP3 group and the low NLRP3 group, respectively. The 10-year survival rates were 55.2 and 78.8% in patients with the high NLRP3 group and the low NLRP3 group, respectively. In summary, high expression of NLRP3 is associated with poor survival in colon adenocarcinoma.

### NLRP3 served as an independent risk factor for the prognosis of colon adenocarcinoma

**Figure 4:**
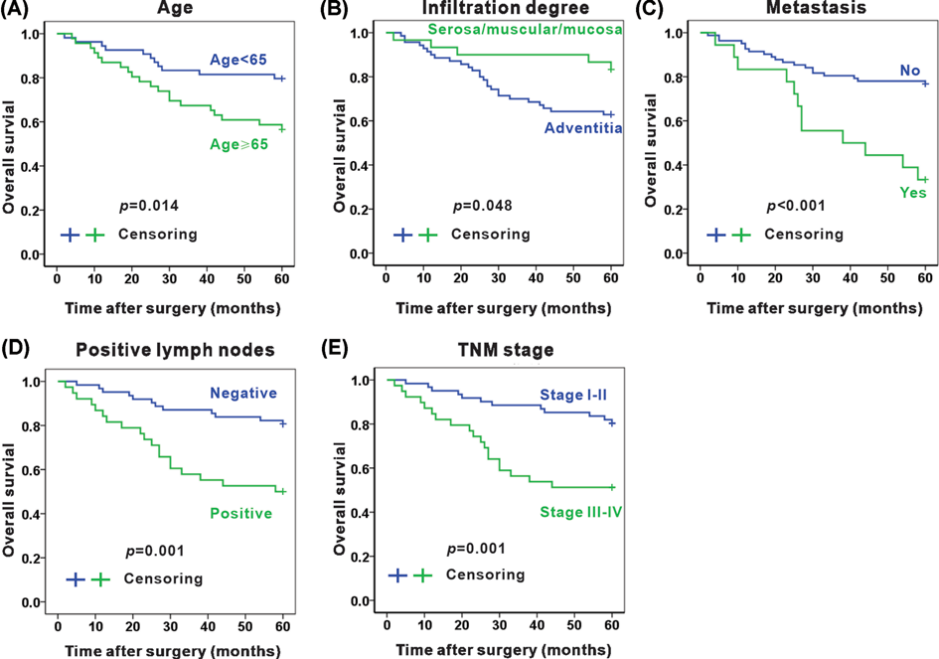
Association between clinicopathological factors and 5-year overall survival in colon adenocarcinoma patients Association between 5-year overall survival and age (**A**), infiltration degree (**B**), metastasis (**C**), positive lymph nodes (**D**), and TNM stage (**E**) in colon adenocarcinoma patients, respectively.

**Figure 5:**
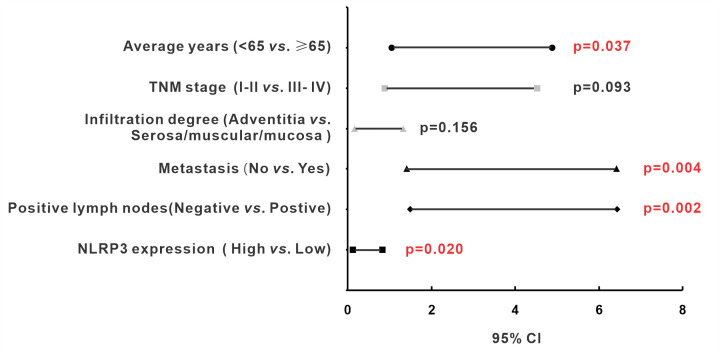
Independent risk factors for prognosis of patients with colon adenocarcinoma The range of 95% confidence interval (CI) and *P*-value presents in the figure.

By Kaplan–Meier survival analysis of clinicopathological characteristics, elder than 65 in age (*P*=0.014), deep infiltration degree (*P*=0.048), the occurrence of metastasis (*P*<0.001), positive lymph nodes (*P=*0.001), and advanced TNM stage (*P=*0.001) were also associated with poor prognosis (Figure 4). While other clinicopathological features, including gender, histological grade, location, and pathological morphology, present no significant correlation with prognosis. Afterward, a multivariable Cox proportional hazards regression model was applied to assess whether these variable factors were independent risk factors for the prognosis of CRC patients (Table 3 and Figure 5). It indicated that high NLRP3 expression (*P=*0.020), elder than 65 in age (*P*=0.037), metastasis (*P*=0.004), and positive lymph nodes (*P=*0.002) were able to predict the poor prognosis of colon adenocarcinoma independently. In contrast, TNM stage and infiltration degree were not the independent risk factor for prognosis in the present study (*P*>0.05). In summary, NLRP3 is an independent risk factor for the prognosis of colon adenocarcinoma.

**Table 3:** Multivariable Cox regression analysis of overall survival in 100 colon adenocarcinoma cases

Features	HR	(95% CI)	*P-*value
Average years (<65 *vs*. ≥65)	2.262	1.049–4.879	0.037
TNM stage (I–II *vs.* III–IV)	2.008	0.890–4.528	0.093
Infiltration degree (Adventitia *vs*. Serosa/muscular/mucosa)	0.473	0.168–1.331	0.156
Metastasis (No *vs*. Yes)	3.007	1.409–6.416	0.004
Positive lymph nodes (No *vs*. Yes)	3.106	1.499–6.435	0.002
NLRP3 expression (High *vs*. Low)	0.321	0.123–0.837	0.020

Abbreviations: CI, confidence interval; HR, hazard ratio.

### Inhibition of NLRP3 suppressed cell proliferation and induced cell apoptosis in HCT116 cells

**Figure 6:**
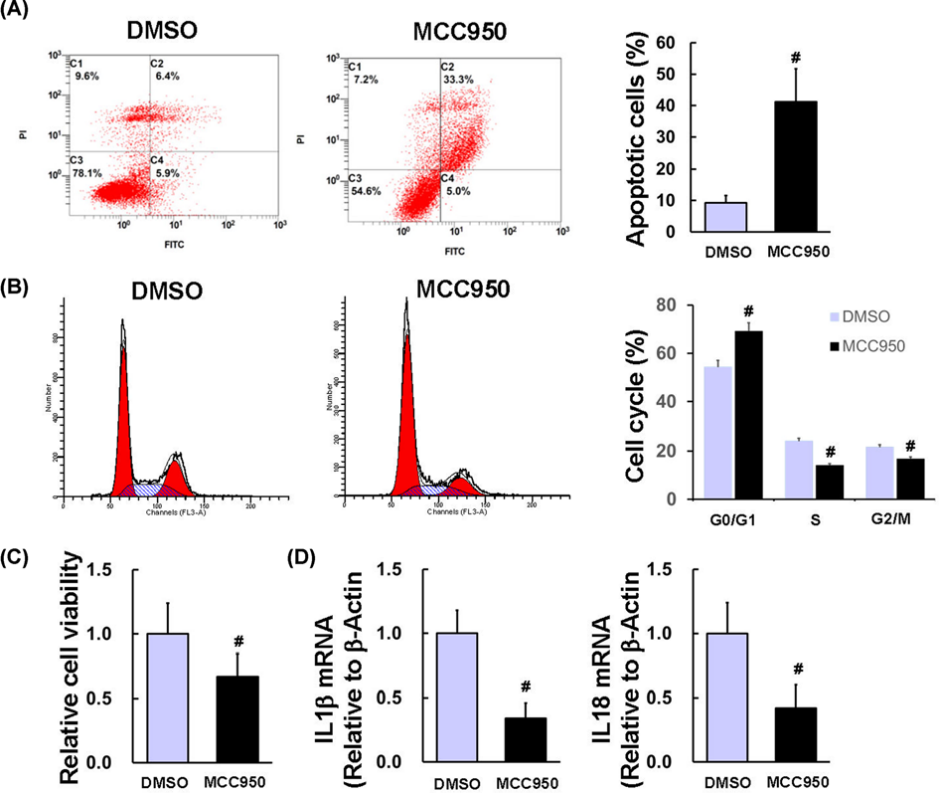
Inhibition of NLRP3 suppressed cell proliferation and induced cell apoptosis in HCT116 cells HCT116 cells were seeded in six-well plates and treated with DMSO or MCC950 (2 μM) for 24 h. (**A**) Cell apoptosis was quantified by flow cytometric analysis of Annexin V-FITC and PI. (**B**) Cell cycle was quantified by flow cytometric analysis of PI. (**C**) Cell viability was determined by CCK-8. (**D**) The mRNA levels of IL1β and IL18 were quantified by qRT-PCR. *n*=3/group, ^#^*P*<0.05 *vs*. DMSO group.

Next, we explored the mechanism of NLRP3 in the regulation of cell proliferation and apoptosis in HCT116 cells. Cell apoptosis was significantly increased after treatment with NLRP3 inhibitor MCC950 compared with DMSO-treated cells (Figure 6A, 41.2 ± 10.5 *vs.* 9.2 ± 2.4%, *P*=0.007). Compared with DMSO-treated HCT116 cells, cells in the G_0_/G_1_ phase were significantly increased after treatment with MCC950, whereas cells in S and G_2_/M phase were significantly decreased after treatment with MCC950 (Figure 6B, *P*<0.05). This anti-proliferation effect by MCC950 was also confirmed by the CCK-8 assay (Figure 6C, *P*=0.02). IL1β and IL18 are downstream of the NLRP3 inflammasome. We next verified the effect of MCC950 on the expression IL1β and IL18 by qPCR. As expected, the mRNA levels of IL1β and IL18 were significantly decreased after treatment with MCC950 (Figure 6D, *P*<0.05). In summary, inhibition of NLRP3 by MCC950 may suppress cell proliferation and induce cell apoptosis via inhibiting IL1β and IL18.

## Discussion

Despite the mass dedication to figure out the pathogenesis and to discover more effective examinations and treatments, the prognosis of CRC patients remains unfavorable. Risk factors of CRC prognosis are now a promising research area, and a set of novel clinical risk factors have been identified, such as excess body weight [21], periodontal disease [22] etc. Some novel molecular markers have been explored to serve as potential predictors for the CRC progression and prognosis [23]. It is undisputed that chronic inflammation would lead to carcinogenesis in the colon. NLRP3 inflammasome leads to potent inflammation and cell pyroptosis in response to DAMPs and PAMPs. However, whether NLRP3 is the independent risk factor for the prognosis of CRC is largely unknown. In the present study, we found that NLRP3 is up-regulated in colon adenocarcinoma tissues of human TMA, HCT116, and RKO xenografts. NLRP3 is an independent risk factor for the prognosis of colon adenocarcinoma. Inhibition of NLRP3 by MCC950 may suppress cell proliferation and induce cell apoptosis via inhibiting IL1β and IL18. These results suggest that the NLRP3 may serve as a putative prognostic biomarker and a potential therapeutic target in CRC treatments.

The role of NLRP3 in the development of CRC is controversial. It is reported that NLRP3^−/−^ knock-out mice showed increased acute and recurring colitis and colitis-associated cancer in the DSS and azoxymethane + DSS models, indicating NLRP3 as a tumor suppressor gene [13]. However, contradictory results also indicated that NLRP3 might function as an oncogene. For instance, lack of NLRP3 in mice significantly attenuated tumor burden than control wild-type mice via an NK cell and IFN-γ-dependent manner [24]. In consequence, the exact effects of NLRP3 upon tumorigenesis and prognosis remain to be elucidated. In the present study, we novelty applied the human TMA cases of CRC patients to determine the NLRP3 expression in colon adenocarcinoma tissues of a human. It was found that NLRP3 expression was significantly up-regulated in colon adenocarcinoma tissues compared with corresponding paracancerous tissues, which is consistent with that in xenograft mice. Since NLRP3 has been considered a more critical role in tumorigenesis and cancer progression, it has not been regarded as a potential diagnostic index and prognosis predictor. NLRP3 was found up-regulated in various forms of cancer such as lung cancer, lymphoma, oral squamous cell carcinoma etc. [25–27], indicating its potential role in the adjuvant diagnostic index. The up-regulation of NLRP3 in colon cancer may attribute to the activation of NFκB and TGFβ signaling pathways [28]. Compared with previous clinical or animal researches concerning NLRP3, we are the first to apply survival analysis and multivariable Cox proportional hazards regression model to evaluate the prognostic effect of NLRP3 in colon adenocarcinoma and discovered a novel result that NLRP3 is an independent risk factor for the prognosis of CRC patients.

Although physiological inflammation was beneficial to the intestine, overwhelming inflammatory microenvironment and excessive inflammatory cytokines can ultimately prompt colonic tumorigenesis [29,30]. As a critical initiator of inflammation, NLRP3 inflammasomes play a critical role in regulating the inflammatory microenvironment by inflecting innate immune responses, recruiting immunocytes, mediating cell deaths, and stabilizing gut microbiota [31,32]. The specific effect varies with the signal pathways involved. Activation of the IL18 pathway shows a protective effect in CRC [33]. While the IL1 pathway mainly activated by NLRP3 inflammasomes contributes to the progression and metastasis of breast cancer [34]. Moreover, activation of NLRP3 inflammasomes promoted tumor metastasis of patients with mammary carcinoma [35]. In the present study, positive correlations were established between NLRP3 expression and TNM stage, distant metastasis and vascular invasion, and positive lymph nodes. Also, MCC950 suppresses cell proliferation and induces cell apoptosis, decreases IL1β and IL18. Therefore, it is presumable that excessive colorectal inflammation promotes the progression of CRC via up-regulation and activation of NLRP3 inflammasomes— IL1β/IL18 signaling pathway. However, further studies are needed to manifest the molecular and cellar mechanism of how increased NLRP3 promotes carcinogenesis of CRC.

Immunocytes are the dominant defensive force against a tumor. Consequently, the survival of cancer patients hinges upon the viability of immunocytes. In the present study, high expression of NLRP3 correlated with poor 5- and 10-year survival in colon adenocarcinoma. Predictably, increased NLRP3 expression might harm the survival of CRC patients by impairing the antitumor function of immunocytes. Increased expression of NLRP3 enhanced the induction and expansion of myeloid-derived suppressor cells (MDSCs) to the tumor microenvironments and resulted in suppression of antitumoral effects of T cells [36,37]. By abrogating NLRP3-dependent MDSC accumulation, NLRP3^−/−^ knock-out mice showed four-fold more prolonged survival than wild-type mice [36]. Furthermore, NLRP3 promotes carcinogenesis and metastases by inhibiting the antitumor effect of NK cells via activating the IL1β–IL1R signaling pathway [24]. It has been revealed in the present study that up-regulation of NLRP3 expression was found both in immunocytes and CRC cells in human CRC patients and HCT116 and RKO xenografts. These pieces of evidence confirm our hypothesis that immune micromovement might contribute to the poor prognosis of CRC patients with high expression of NLRP3.

In conclusion, high NLRP3 expression predicts poor progression and survival in colon adenocarcinoma. NLRP3 is an independent risk factor for the prognosis of CRC patients. Inhibition of NLRP3 by MCC950 may suppress cell proliferation and induce cell apoptosis via inhibiting IL1β and IL18. Our results shed light on the role of NLRP3 as a putative prognostic biomarker of colon adenocarcinoma and a potential therapeutic target in CRC treatments.

## Data Availability

All the data are present in the manuscript. All the data are available from the corresponding author Jinhang Gao (jinhang@wchscu.cn or Gao.jinhang@qq.com) under reasonable request.

## Abbreviations

ASC: apoptosis-associated speck-like protein containing a C-terminal caspase recruitment domain
CCK8: cell counting kit-8
CRC: colorectal cancer
DAMP: damage-associated molecular pattern
DSS: dextran sulfate sodium
GAPDH: glyceraldehyde 3-phosphate dehydrogenase
GSDMD: Gasdermin D
H&E: Hematoxylin–Eosin
IHC: immunohistochemistry
IL18: interleukin 18
IL1β: interleukin 1β
IOD: integral optical density
MDSC: myeloid-derived suppressor cell
NLRP3: NOD-like receptor pyrin domain-3
PAMP: pathogen-associated molecular pattern
PI: propidium iodide
TMA: tissue microarray
TNM: TNM classification of malignant tumors
